# Research developments in nanobiomaterials for tumor vaccines: A review

**DOI:** 10.1097/MD.0000000000050321

**Published:** 2026-08-28

**Authors:** Hengheng Dai, Weiwei Kong, Tingting Liu, Xiaoxia Fei

**Affiliations:** a Department of Pharmacy, Yancheng Third People’s Hospital, Affiliated with the Yancheng School of Clinical Medicine of Nanjing Medical University, Yancheng, Jiangsu Province, China; b The Comprehensive Cancer Center of Nanjing Drum Tower Hospital, The Affiliated Hospital of Nanjing University Medical School, Nanjing, Jiangsu Province, China.

**Keywords:** biomimetic nanomaterials, immunotherapy, inorganic nanomaterials, lipid nanomaterials, polymeric nanomaterials, tumor vaccines

## Abstract

One of the most researched forms of tumor immunotherapy is tumor vaccines, which activate antigen-presenting cells (APCs) by administering adjuvants and tumor antigens, triggering an immune response against the tumor. Tumor growth is remarkably inhibited, and tumor metastasis and recurrence are prevented by tumor vaccines. However, to increase therapeutic benefit and ultimately improve the clinic, it is imperative to identify efficient delivery materials and mechanisms, as conventional tumor vaccines have shortcomings, such as low potency of their encapsulated antigens and a low ability to reach APCs. Numerous nanobiomaterials have the potential to significantly increase the efficacy of tumor vaccination, enhance their anti-tumor properties, and reduce the consequences of off-target effects. To promote the progress of nanobiomedicine in tumor immunotherapy and provide new perspectives for the design and implementation of tumor vaccine platforms, this article takes an in-depth look at the development of nanobiomaterials, including polymeric, lipidic, inorganic, and biomimetic nanomaterials.

## 1. Introduction

Globally, cancer poses a significant threat to human life and health: 19.29 million new cases and 9.96 million cancer deaths were reported in the GLOBOCAN data for 2020.^[1]^ After being awarded the Nobel Prize in Physiology or Medicine in 2018, tumor immunotherapy has steadily gained importance as a therapeutic cancer treatment method. It does this by initiating and maintaining the tumor-immune cycle, boosting the immune system, identifying and eliminating aberrant cells, and ultimately reducing and controlling tumor growth.^[2]^

Tumor vaccines are a type of immunotherapy that activate T cells in vivo by delivering tumor antigens and immunoadjuvants to lymph nodes (LNs). The LNs are then taken up and processed by antigen-presenting cells (APCs), which release the antigens and present them to the T cells. This leads to the induction of long-lasting immune memory and a sustained and effective tumor-specific immune response that inhibits tumor growth, recurrence, and metastasis.^[3,4]^ Tumor antigens, immunological adjuvants, and delivery vectors make up the majority of the active components of tumor vaccines.^[5]^ Shortcomings of conventional tumor vaccines such as DNA, mRNA, and dendritic cell (DC) vaccines include the limited efficacy of their encapsulated antigens and a low ability to reach APCs. Consequently, there is an urgent need to develop safe and efficient delivery methods and materials to improve the efficacy of vaccination. With the rapid development of materials science and nanotechnology, nanobiomaterials have gained more and more attention.

Since it is frequently not possible for neoantigen vaccines based on peptides or nucleic acids to target or reach lymph node DCs, the use of nanoparticles (NPs) has long been seen as an appealing means of enhancing vaccine delivery. Nanovaccines can be efficiently transported to secondary lymphoid tissue and kept in lymph nodes for a considerable amount of time thanks to the nanovaccine delivery method. Second, several synergistic adjuvants and antigens can be co-delivered to lymph nodes and APCs via nanovaccines. Furthermore, nanovaccines can enhance the pharmacokinetic characteristics of their therapeutic payloads by co-encapsulating adjuvants and antigens, hence amplifying the subsequent immune regulatory effects.^[6-8]^ In recent years, the novel approach of combining tumor vaccines with nanotechnology has attracted much attention. Through electrostatic, hydrophobic, hydrogen bonding, covalent bonding and other interactions, nanobiomaterials can effectively encapsulate adjuvants and antigens in nanovaccine platforms. They can also be used to target lymph nodes and modify the interactions between vaccines and APCs by altering their surface properties, such as particle size, surface charge, morphology and hydrophilicity.^[9]^

There are many different types of nanomaterials available today. In a review of the relevant literature, we found that polymers, lipids, inorganics, and biomimetic nanomaterials are most commonly used for tumor vaccines. In an effort to help researchers select safe, practical and affordable nanomaterials for the development and application of tumor vaccines, we have compiled the research on the above-mentioned nanomaterials in this section.

## 2. Polymer nanomaterials

Numerous polymers have antigen storage effects, APC targeting, immunomodulation and other properties. These include natural polymers, block copolymers and dendrimers. The high safety of these polymers, their good stability and simplicity of surface modification have led to reports of their use as vaccine carriers.

### 2.1. Natural polymers

Chitosan and HA polysaccharides, 2 polymers produced by living organisms, are widely used for vaccine delivery due to their biocompatibility, adaptability, and low immunogenicity. Chitosan is the only positively charged polysaccharide due to the amine groups in its backbone, which also allow it to bind more easily to various chemicals or biomolecules. To act as an antigen carrier, Pei first added catechol groups to chitosan and created chitosan/calcium phosphate nanosheets by immersing it in Na_2_HPO_4_ solution and then directly combining it with CaCl_2_.^[10]^ In vitro experiments showed that the antigen carrier nanosheets improved the efficiency of absorption of exogenous antigens and retention in the lymph nodes after antigen loading by nanoprecipitation, with 98.7% of antigens reaching the cytoplasm. In addition, a significant increase in antigen cross-representation was observed by the nanosheets compared to free antigen. Cao’s group used polymeric nanoparticles modified with the natural anionic polysaccharide HA and showed that these are promising to enhance humoral and cellular immune responses. The work shows how to modify the sulfhydryl groups of OVA with the Traut reagent, combine them with Au NPs, and then link them to HA to produce NIR-responsive nanovaccines. Smooth muscle cells, tumor cells, and DCs all express the HA receptor CD44, which enables DCs to efficiently endocytose HA-modified AuNP nanoparticles and increase antigen uptake. In mice with EG.7-OVA tumors, the experimental group showed significant inhibition of tumor growth and prolonged survival.^[11]^

### 2.2. Block copolymers

Chain segments are used to combine 2 or more polymers with different properties to create block copolymers. The monomers lactic acid and hydroxyglycolic acid are polymerized in a random ratio to produce the polylactic acid-hydroxyglycolic acid copolymer (PLGA), which has good film-forming, capsule-forming, and biocompatible properties. Due to the advantages of the polyester materials polylactic acid (PLA) and polyglycolic acid (PGA), it is widely used in biomedical engineering. The US FDA has approved the sale of more than a dozen PLGA microsphere formulations.^[12]^

Researchers have developed Pickering emulsion-based self-adjuvanted nanovaccine systems (PPAS) to enhance antigen adsorption. PPAS uses PLGA nanoparticles as colloidal stabilizers and squalene as the dispersion phase. Studies show that PPAS facilitates the release and dispersion of antigens at the injection site, increasing CD86 expression on DC surfaces and resulting in a survival rate of 87.5% in mice with melanoma.^[13]^ Liu prepared nanoparticles (HA-DOTAP-PLGA NPs) loaded with ovalbumin using a biphasic emulsion and solvent evaporation technique. The NPs increased cytokine and co-stimulatory molecules, and in vivo studies showed stronger antigen-specific CD4^+^ and CD8^+^ T cell responses in NP-immunized mice, suggesting they could enhance immune responses.^[14]^ In 2019, Connint modified the mannose on the surface of PLGA to create biodegradable nanoparticles loaded with toll-like receptor agonists and melanoma-expressed antigenic peptides. Studies showed that immunized mice activated immune cells to kill melanoma cells, suggesting that mannose-modified nanomaterials can efficiently target melanoma cells, as the mannose receptor (MR/CD206) was abundantly expressed in DCs.^[15]^

Amphiphilic polymers can be synthesized using reversible addition-rupture chain transfer polymerization (RAFT) or free radical atom transfer polymerization (ATRP), resulting in nanomicelles with hydrophobic active ingredients for in vivo applications. Block copolymers with numerous amino groups on their side chains activate the complement system, making them suitable as vaccine adjuvants.^[16,17]^ Pan developed a triblock polymer (TPCAH) by synthesizing mPEG_5k_-PAGE15 diblock copolymer, mPEG_5k_-PAGE15-PCL_5k_ and mPEG_5k_-PAGE15(NH_2_)-PCL_5k_. This positive charge can electrostatically combine with OVA to form polymeric nanoparticles that enhance the vaccine’s immunological response. In vitro cellular studies showed that TPCAH@OVA has no significant cytotoxicity and can be efficiently endocytosed by APCs, promoting lymphocyte proliferation and stimulating cytokine production.^[18]^A team created a cationic pentablock copolymer based on Pluronic F127, an FDA-approved injectable thermogel-polyoxyethylene-polypropylene-vinyl triblock copolymer, using an ATRP reaction with N-propyl pyridylimine (NPPM) ligand, copper oxide nanoparticles, and a macromolecular initiator of Pluronic.^[19]^ A study injected reagents into mice’s abdomens, and 30 days later, no inflammation or damage was found. However, subcutaneous injection of Pluronic F127 increased blood cholesterol and triglyceride concentrations. To mitigate this, the vaccine adjuvant formulation was modified by adding polyvinyl alcohol, allowing gelation and reducing the amount of Pluronic in the formulation.

### 2.3. Other polymers

Fluorinated polymers, such as fluorinated alkyl-grafted polyethylenimines (F-PEIs), have the potential to serve as protein sources due to their self-assembly properties and low surface energy. These fluoroalkyl chains are both oleophobic and hydrophobic, with hydrophobicity enhanced by perfluoroalkyl side chains.^[20]^ Grafting cationic polymers with fluorinated alkyl chains can enhance their ability for transmembrane delivery of drugs or antigens into cell lysates.^[21,22]^ F-PEIs were used by Xu as an antigen carrier. In combination with OVA, they self-assembled into F-PEI/OVA NPs. This fluorine-containing preparation effectively inhibited B16 OVA tumors in vivo by eliciting a high level of antitumor T cell responses.^[23]^ Qin developed a thermoreactive gel nanovaccine using polyamide dendrimer (PAMAM), a flexible organic macromolecule with a nanoscale dendritic structure. The gel system encapsulates reductively cleaved PAM chains, adriamycin, CpG, and the photothermal agent indocyanine green. The heat of the laser irradiation leads to apoptosis of the tumor cells, activates the nanoplatform, and releases CpG and DOX. This increases the effectiveness of antigen cross-presentation.^[24]^

## 3. Lipid

Liposomes are vesicles with a hydrophilic head and a hydrophobic tail consisting of lipid bilayers. They have controlled release properties, good biocompatibility, and are easily absorbed by tumor cells. Liposomal nanobiomaterials (LPX) are mainly composed of lipids, including cholesterol, phospholipids, cationic lipids, and polyethylene glycolized lipids. By encapsulating hydrophilic or hydrophobic antigens between the layers, LPX enable the formation of multilayered structures in liposomes. To regulate the size, polydispersity, and lamellar structure of lipid-based nanobiomaterials, processes such as sonication, extrusion, homogenization, and freeze-thaw cycles have been used.

### 3.1. Cholesterol

Cholesterol promotes vesicle stability by filling the spaces between lipids and within the liposome membrane due to its rigidity and hydrophobicity. Darrell Irvine and colleagues have created nanovaccines by simply conjugating and assembling cholesterol, neoantigenic peptides, and a CpG adjuvant. They used the ability of liposomes to bind to the body’s own albumin and transport the antigen specifically to the lymph nodes. In animal experiments, the resulting amphiphilic CpG DNA/peptide vaccine showed significantly improved anti-tumor activity and a better safety profile than the parent drug.^[25]^ The results of the experiments showed that the tumor foci of the tumor-bearing mice decreased significantly, proving that combination immunotherapy can be used to treat tumor foci. Two years later, the team combined this amphiphilic nano-vaccine with an extended half-life of IL-2, an anti-PD-1 antibody, and an antibody directed against tumor antigens. This shrank the tumors, proving that combination immunotherapy can successfully treat the majority of tumors that are considered refractory in an experimental context.^[26]^ Researchers have developed a tumor vaccine using CpG-coupled HDL cholesterol and an antigenic peptide “nanodisk” structure. The nanoparticles enhanced the co-delivery of CpG and neoantigenic peptides to lymph nodes, enabling DC maturation and a 47-fold increase in neoantigen-specific cytotoxic T cells (CTLs). In combination with anti-PD-1 and anti-CTLA-4, the nanodiscs successfully resisted MC-38 and B16F10 tumor constructs, providing a broad approach for tailored nanomedicine.^[27]^

### 3.2. Cationic lipids

Cationic lipid-based nanobiomaterials are one of the nanoplatforms used for targeted delivery of antigen-encoding mRNAs for LNs by functionalizing the molecular ligands to ensure target cell recognition. Cationic lipids were the first lipid nanoparticles to be used for simple binding to negatively charged nucleic acids.

A cationic lipid complex (SLP–LPX) was created by Arbelaez to target the unique antigen peptide linked to the KRAS G12D mutation. The combination of the SLP–LPX vaccine with ICIs was also found to dramatically reduce tumor growth. Following immunization, it can boost CD4^+^ and CD8^+^ T cells and suppress tumor growth in a manner that is dependent on CD8^+^ T cells.^[28]^ Heuts discovered that cationic liposomes of DOTAP and dioleoylphosphatidylcholine (DOPC) can effectively load and deliver synthetic long peptides (SLPs) specific for the epitope of SIINFEKL T cells to DCs. This activation of SIINFEKL-specific CD8^+^ T cells then mediates tumor regression.^[29]^

### 3.3. 1,2-distearoyl-sn-glycero-3-phosphoethanolamine-polyethylene glycol

The conjugation of polyethylene glycol (PEG) with 1,2-Distearoyl-sn-glycero-3-phosphoethanolamine (DSPE) leads to DSPE–PEG, an amphiphilic liposome that exhibits both hydrophilic and hydrophobic properties. PEG is often used to improve the properties of phospholipids. The end of DSPE–PEG can be modified to allow the binding of ligands such as nucleic acids or peptides. To create DSPE–PEG–NHS, a team of researchers attached active esters to the end of the PEG chain. The NHS moiety was then used to conjugate with amine-containing molecules to enable lymph node-targeted delivery of adjuvants and neoantigenic peptides in lipid bilayers.^[30]^ It was shown that mice given nanovaccines had a 10-fold increase in neoantigen-specific T cells and increased levels of tumor necrosis factor-alpha (TNF-α) and interferon-gamma (IFN-γ) compared to mice given free vaccines. The researchers developed an active LN-targeting nanovaccine (ALAS) by modifying the surface of lymphatic endothelial cells by incorporating the targeting moiety, the azide group (N_3_), into the molecular endpoint of DSPE–PEG. This modification allows the OVA peptide and the TLR agonist poly (I:C) to be readily encapsulated in liposomes, leading to tumor suppressive effects in subcutaneous melanoma models, prolonging the survival of mice with tumors, and promoting antigen and adjuvant aggregation in lymph nodes.^[31]^

## 4. Inorganic nanomaterials

Inorganic nanoparticles have attracted increasing attention with the advances in nanotechnology due to their significant therapeutic potential for cancer.

### 4.1. Silicon dioxide

Dong prepared nanovaccines (OVA@ SiO_2_) by covalently modifying OVA on the surface of SiO_2_ nanospheres using the Stober method.^[32]^ Experiments showed that the produced nanovaccines exhibited good biocompatibility with important organs and a high antigen loading capacity. In addition, OVA@ SiO_2_ stimulates DC2.4 cells to take up antigen and release it into the cytoplasm, where it can be used as a carrier for antigen delivery.

It has been observed that mesoporous silica nanoparticles (MSNPs) possess inherent immunogenicity.^[33]^ Through surface modification techniques such as adsorption, encapsulation, and coupling of functional groups, MSNPs can lower their surface energy and reduce their tendency to aggregate. To create an antigen complex, Zhou developed a nanovaccine carrier system with dual reactivity in terms of pH and reducing properties. First, polyethylenimine (PEI) was adsorbed onto metal polyphenol networks (MPNs) as a nanocarrier. Then, OVA was added to the MSNPs by electrostatic adsorption, and in the end, the system was covered with metal-polyphenol networks (MPNs) containing a large number of disulfide bonds sensitive to reduction. By utilizing the proton sponge effect of PEI, the nanovaccine enhanced the lysosomal escape efficiency of the antigen and additionally released it into a reducing medium that resembled the cytosol. The subcutaneous PMSN@OVA-MPN nanovaccine enhanced the immunological memory effect, accelerated the marked proliferation of CD8^+^ T cells, and strengthened MHC-I-mediated cellular immunity.^[34,35]^

### 4.2. Other metals

Gulla was the first to produce gold nanoparticles modified with thiol ligands to develop DNA-based tumor nanovaccines (Au-SGSH-DNA).^[36]^ Au-SGSH NPs were able to deliver the DNA vaccine to the DCs of mice after subcutaneous treatment in vivo. This triggered a long-lasting immune response that inhibited melanoma growth and increased the survival rate of the animals. Luo’s team has shown that iron oxide nanoparticles (Fe_3_O_4_ NPs) can be used as novel immune enhancers. Fe_3_O_4_ NPs were combined with OVA to produce a novel nanocomposite vaccine. The potential of iron-based nanomaterials for clinical applications is enhanced by the fact that Fe_3_O_4_ NPs enabled the Fe_3_O_4_-OVA vaccine to successfully prevent and inhibit the formation and development of subcutaneous and metastatic tumors, activate T cells and macrophages, and stimulate the maturation of bone marrow dendritic cells (BMDCs).^[37]^Manganese nanoadjuvants (MnJ) function as both immune activators and delivery systems, and some studies suggest that Mn^2+^ or preparations have cellular immunoadjuvant effects.^[38,39]^ By administering 3 doses of MnJ-OVA to mice before immunizing them subcutaneously with B16-OVA melanoma cells, the researchers evaluated the adjuvant effect of manganese salts on tumor vaccines. They found that this significantly inhibited tumor growth and increased intratumoral infiltration of activated NK cells and CD8^+^ T cells.^[40]^

## 5. Bionanomaterials

The development of multifunctional nanomaterials derived from biomimetic materials has been used to improve the safety and efficacy of nanovaccines. In numerous research studies, various biomimetic nanomaterials, including membrane-welded nanomaterials, artificial APCs, naturally derived nanomaterials, and tumor cell-derived nanomaterials, have been used as natural mimetic systems for tumor vaccine delivery.

### 5.1. Naturally derived nanomaterials

Nanobiomaterials derived from natural sources may have superior efficacy in enhancing immune responses against tumors and mitigating harmful side effects. To date, a variety of naturally occurring nanobiomaterials have been thoroughly investigated, including albumin, exosomes, erythrocytes, and outer membrane vesicles (OMVs).

#### 5.1.1. Erythrocyte membrane

Human peripheral blood has a large number of easily accessible erythrocyte cells, and erythrocyte membranes can be used as natural carriers for the targeted administration of vaccines. The main organ targeted for vaccination is the spleen, which also eliminates and scavenges damaged erythrocytes. To preserve the biological properties of the erythrocyte membranes and improve their ability to transport drugs, the researchers added mannosylated lipid materials. They also encapsulated proteins from induced pluripotent stem cells (iPSC) to develop an antitumor nanovaccine (iPSC@RBC-Mlipo) that can specifically stimulate the maturation of splenic APCs to elicit an effective antitumor immune response. The vaccine can achieve both preventive and therapeutic goals by suppressing the growth of primary tumors, fighting metastatic tumors, and successfully preventing tumor recurrence after surgery.^[41]^ Since Listeria monocytogenes (Lmo) is rapidly eliminated from the bloodstream, intravenous administration of the bacteria causes systemic inflammation. Liu’s group has developed a bionic Lmo@RBC to reduce the systemic inflammation caused by intravenous administration of Listeria monocytogenes (Lmo). This method promotes anaerobic Lmo colonization in tumors due to extensive blood circulation and a hypoxic tumor microenvironment. Intravenous injection of PBS, Lmo, or Lmo@RBC induces cellular pyroptosis, which reverses the immunosuppressive tumor microenvironment and stimulates a robust anti-tumor immune response that effectively fights solid tumors and tumor metastases.^[42]^

#### 5.1.2. Exosomes

Exosomes are a type of extracellular vesicle that are nanoscale and possess the right structure and properties to mediate efficient cell–cell interactions. Exosomes have a number of adhesion proteins and carrier ligands on their surface that can attach to target cells and help to precisely transport and release loaded drugs.^[43]^ Reportedly, DCs expressing α-fetoprotein (AFP)-derived exosomes (DEX) can be used as nanovaccines (DEXAFP) to effectively elicit an immune response specific for the antigen.^[44]^ DEXAFP was able to significantly reduce tumor development and prolong survival after intravenous injection into a liver tumor model. This was achieved by improving the immunological microenvironment of the tumor (TIME) and increasing the expression levels of IL-2, IFN-γ, and CTL.

#### 5.1.3. Outer membrane vesicles

A TMV vaccine was created by attaching glycosylphosphatidylinositol (GPI)-anchored versions of immunostimulatory B7-1 (CD80) and IL-12 molecules to tumor cell outer membrane vesicles (OMV) that were recovered from 4T1 tumor tissues.^[45]^ In combination with ICB, mice’s survival was greatly increased, lung metastases were decreased, and immunomodulatory cytokines were upregulated in plasma. Bacterial-derived OMV has been studied as a potential novel adjuvant in recent years.^[46]^ Proteolipids, which are spherical and nanosized and contain outer membrane proteins, are secreted by gram-negative bacteria and can be utilized as carriers or inactive complex vaccines. The study’s findings indicate that OMV specifically targets and accumulates in tumor tissue, where it is followed by the antitumor cytokines IL-12p40, CXCL10, and IFN-γ secretion that induces a long-term antitumor immune response. The study also measured the fluorescence intensity of OMV throughout the body of Dutch CT26 mice after administering the fluorescently labeled OMV and observing the highest intensity of fluorescent signal in tumor tissue.^[47]^ Bacterial extracellular vesicles have the potential to target tumors because of their nanoscale size, which allows them to passively target tumor vasculature with specialized permeability through high permeability and retention (EPR effect).

#### 5.1.4. Albumin

Albumin is highly immunogenic, stable, and biocompatible. It may be efficiently ingested by APCs by endocytosis, which makes it possible to distribute vaccines for antigen cross-presentation in an efficient manner. The vaccine molecule attaches to albumin in the circulation and hitches a ride to the lymph nodes using albumin’s “hitchhiking” approach. Based on this approach, Kim created a hydrophilic and lipophilic amphiphilic vaccine by using the transporter proteins of albumin’s fatty acid molecules to extract protein fragments that attach to albumin and by creating self-assembling nanomembranes by adding lipid tails to the vaccine peptide and CpG adjuvant (lipo-CpG) . According to the findings, the vaccination improved anti-tumor activity in the TC-1 and B16F10 tumor models and aggregated in LNs. Comparing the systemic toxicity to the parent chemical, it was significantly lower.^[25]^

#### 5.1.5. Gels

Gels are a type of nanobiomaterial that improves tumor vaccination by releasing adjuvants and antigens constantly, focusing on particular immune cells, extending in vivo bioactivity, and promoting strong and durable immune responses.^[48]^ In the early stages of research, Park created hydrophobic transforming growth factor-β (TGF-β) inhibitors and biodegradable nano-liposome polymer gels (nLGs) for co-delivery of IL-2. Through the EPR effect, this technique effectively enriched tumor tissues by increasing the solubility of TGF-β inhibitors and reducing their clearance by the reticuloendothelial system during blood circulation. TGF-β inhibitors and IL-2 were released by nLGs, which markedly slowed down the growth of tumors, prolonged the lives of mice harboring tumors, and enhanced the infiltration of CD8^+^ T cells and NK cells inside the tumor.^[49]^

### 5.2. Tumor cell-derived nanomaterials

Numerous autologous tumor materials containing high concentrations of tumor-associated antigens (TAAs) have been developed as platforms for nanovaccines that can effectively elicit multi-antigen anti-tumor immune responses, based on the concepts of biomimetic nanotechnology. Because encapsulating cell membranes gives nanoparticles (NPs) excellent biocompatibility and adjustable surface characteristics, cell membrane-encapsulated biomimetic carriers are highly promising for use in cancer treatment.

Wu suggested encapsulating magnetic Fe_3_O_4_ nanoparticles in HepG2 hepatocellular carcinoma cell-derived cell membranes to create tumor cell membrane-encapsulated Fe_3_O_4_@SiO_2_ magnetic nanoparticles (CMNPs), which are based on cell membrane biomimetic magnetic nanoparticles. This step would increase the biocompatibility, stability, and dispersion of the magnetic nanoparticles. Enhancing NK cell-mediated anti-tumor effects against HepG2 cells, functionalized nanoparticles containing tumor-specific antigens on their surface upregulated the expression of activation receptors and markers on the surface of NK cells. This, in turn, induced the secretion of cytotoxic factors, such as perforin and granzymes.^[50]^ To create a customized tumor vaccine (CpG-CCNP), Kroll created NPs with CpG adjuvant encapsulated in PLGA, which were then coated with melanoma cell membranes placed on the NPs. The vaccine was administered to mice in the B16F10 tumor model; the mice accumulated in the draining lymph nodes, facilitated antigen cross-presentation, and generated elevated pmel-1 T-cell proliferation.^[51]^ Ochyl employed an easy-to-use yet efficient therapeutic vaccination technique using autologous melanoma cell lysates. To create PEG NPs, tumor cell membranes underwent a series of treatments including trypsinization, freeze-thaw, sonication, and PEGylation. PEG NPs had good systemic stability in vitro.^[52]^ PEG NPs were demonstrated to have high lymph node drainage efficiency and the ability to elicit antigen-specific CD8^+^ T cell responses following subcutaneous injection.

### 5.3. Artificial APCs

An exploration of immune cells with nanomaterials is done with APC mimics. Artificial APCs based on nanomaterials (aAPCs) imitate the role of genuine APCs in conveying antigenic signals to T cells, inducing tumor-specific immune responses without requiring delivery of adjuvants and antigens that activate the natural APCs.^[53]^ APCs have regulated biological behavior and a composition that is comparatively well-defined. pMHC, T-cell receptor ligands, co-stimulatory receptor ligands, and cytokines like ILs and IFNs are typically added to the surface of biomimetic nanobiomaterials to modify the biomimetic nanovaccines generated from aAPCs.^[54,55]^

An aAPC vaccine was created very early on using latex microspheres coated with anti-CD28 antibody, 4-1BB ligand, H-2Kb-Ig/pTRP2 dimerization complex, and CD83 molecule.^[56]^ Melanoma-specific CTLs were activated by intravenous infusion of the aAPC vaccination in a mouse model of melanoma. While TRP2-expressing H-2K^(b+)^ melanoma cells and RMA-S lymphoma cells pulsed with the peptide pTRP2 were specifically cleaved by generated CTLs, the murine Lewis lung cancer cell line 3LL eluded the CTLs. Zhang used a click chemical reaction to load anti-CD28 antibody and pMHC-I onto the surface of iron oxide magnetic nanoclusters (MNCs) after coating them with a layer of N_3_ modified white blood cell membrane. The vaccine was amplified and co-cultured with the original CD8^+^ T cells after being modified using DBCO groups. CTLs were stimulated by the produced nanovaccine (N_3_-LMNC) in vitro, and in vivo CTL migration to the tumor site was noted. In the EG-7 tumor-bearing mice model, it also demonstrated a good effect of slowing tumor growth.^[57]^ In order to create a bionic aAPC-derived nanovaccine, Cheng’s group used DCs membrane-coating nanotechnology to extrude a membrane carrier of stimulated mature BMDCs with IL-2-loaded PLGA nanoparticles. This method preserved the DCs’ capacity for antigen cross-presentation and T-cell stimulation while also promoting increased T-cell activation in vitro and in vivo. When this micro-DC vaccination was used instead of the DC vaccine in a mouse model of ovarian cancer, it decreased tumor metastasis and delayed tumor growth.^[58]^

### 5.4. Membrane fusion nanomaterials

One promising approach to creating biomimetic cellular vaccines is the fusion of autologous tumor cells with immunological or bacterial cells. On the one hand, APCs can ingest certain antigens produced on tumor cells’ membranes to trigger a CTL response. However, OMVs generated from bacteria are rich in adjuvant components that are passed down from the parent bacterium and can be utilized to stimulate the maturation of immune cells.^[59]^

Antigen-laden erythrocyte nanobodies were created by fusing erythrocyte membranes with tumor cell membranes loaded with TAAs using sonication and extrusion, in response to our earlier discussion that injured erythrocytes have the ability to target splenic APCs. A variety of immune cells, including DCs, NK cells, B cells, CD4^+^ T cells, and CD8^+^ T cells were activated when the spleen was targeted in vivo. Th1 and NK also significantly increased the number of cytokines they released. When combined with aPDL1, it greatly reduced the formation of tumors in the B16F10-Luc tumor model; about 44% of the loaded mice lived for more than 40 days.^[60]^

In order to successfully combine melanoma antigens with natural adjuvants for immunotherapy, Chen created eukaryotic-prokaryotic fusion vesicle (EPV) nanovaccines. These were created by combining attenuated Salmonella OMV and B16F10 melanoma cell membrane vesicles (CMVs) through sonication and extrusion. Furthermore, by initially encapsulating the core of PLGA and indocyanine green (ICG) within the nanovaccine as PLGA–ICG NPs, EPV nanovaccines can be further functionalized. By absorbing near-infrared light and transforming it into heat that targets and kills tumor cells, causing immunogenic cell death (ICD) and creating tumor antigens in situ for immune activation, the synergistic anticancer efficacy as a therapeutic vaccine was demonstrated.^[61]^ Because its membrane components activate DCs, Mycobacterium grassii (M.ph) likewise has little toxicity to humans and animals. Bifunctional fusion membrane nanoparticles (FM NPs) were created by combining autologous tumor cell membranes (TM) and Mycobacterium grassii membrane extract (MM). The FM-NPs were then dissolved in sodium alginate (ALG) solution along with granulocyte-macrophage colony-stimulating factor (GM-CSF), which was injected subcutaneously to form an activated DCs hydrogel in situ. It can significantly accelerate DC maturation, generate sufficient memory effector T cells (T_EM_) in TDLNs, raise the percentage of T_EM_ in tumors, and successfully regulate tumor growth in a 4T1 mouse mammary tumor model.^[62]^

Additionally, DCs and tumor cells have been united to create fused cells (FCs), which can improve LNs’ homing capacity and simplify the presentation of antigen to T cells in cell membrane-derived nanovaccines. Owing to the potent passive targeting characteristic of the metal-organic nanoframework (MOF), Liu combined MOF NPs with FCs to create a MOF@FM nanovaccine. According to experimental data, MOF@FM constantly released antigens generated from cell membranes for DC maturation and CTL activation, in addition to demonstrating a strong ability to target LNs. Mice injected with MOF@FM exhibited a high proportion of CTLs (14.5%) and a 3-fold and 27-fold increase in IL-6 and IFN-γ production, respectively, as compared to untreated controls.^[63]^ Zhang created a co-delivery nanosystem of antigen and adjuvant (DTFCsM@PLGA) by encasing poly (lactic acid hydroxyacetic acid; PLGA) microspheres loaded with vaccine adjuvant CpG-ODN using an FC membrane. The study’s findings show that DTFCsM@PLGA It has a significant therapeutic effect on mouse allogeneic transplantation and metastasis models because it possesses both the immunogenicity of tumor cells and the antigen presentation ability of DC. Additionally, it can stimulate the production of a large amount of CTL and promote the infiltration of immune cells at the tumor site.^[64]^

## 6. Article summary

The bulk of traditional tumor vaccines are still in the experimental research phase and are not yet suitable for clinical use because of their numerous shortcomings, which include inadequate targeting, rapid extracellular breakdown, difficult preparation methods, and high cost. Polymers, lipids, inorganics, and different biomimetic nanomaterials all have a unique role to play in improving the delivery of tumor vaccines as nanomaterials technology develops. Since polymeric nanoparticles may be engineered to respond to specific stimuli like bases, acids, or enzymes, vaccine components can be released when needed. Moreover, by adding bioactive substances or cell-targeting ligands to polymers, multifunctional nanovaccines can be produced. Lipid-based nanomaterials offer the highest likelihood of facilitating the large-scale manufacturing of nucleic acid nanovaccines due to their simple composition and proven production methods. A further area of interest for researchers is biomimetic nanovaccines made from autologous cellular components with unique functions since exogenous nanobiomaterials have intrinsic immunogenicity that might cause off-target damage. By modifying cell membranes biomimetically, nanodelivery systems can become more biocompatible and possess sophisticated biological properties like extended circulation, immune evasion, homologous targeting, and so on. This can lead to the enrichment of payloads at the sites of disease and an increase in the effectiveness of treatment. In contrast to single-cell membranes serving a particular purpose, hybrid membranes (HM) including multiple membrane components can include properties from many cells to accomplish a wider range of delivery goals. These can provide us with important data that will aid in the development of better tumor nanovaccines that could find application in medicine.

A search of the National Institutes of Health (NIH) clinical trial website in the United States turned up information about over 7000 cancer vaccines that are presently in the development stage. These include 640 mRNA vaccines, 197 vaccines involving novel antigens, and 7 mRNA vaccines involving immune regulation that are going into clinical practice. The majority are enrolled in phase I/II clinical trials. Currently, the novel antigen cancer vaccine is undergoing or has finished more than 100 clinical trials. However, these therapeutic vaccine trials did not show significant clinical benefits until 2 recent trials (NCT03897881 for melanoma and NCT04161755 for pancreatic cancer) exhibited prolonged recurrence-free survival (RFS).

To sum up, tremendous advancements have been made in the technologies that identify novel antigens and initiate potent new antigen-specific anti-tumor T cell immunity. Still, a lot of challenges need to be solved before vaccinations can effectively prevent cancer. In order for neoantigen vaccines to fulfill their full potential and offer clinical advantages to cancer patients, the domains of tumor vaccine administration and cancer immunology must come up with creative answers to these problems. Although there are still many challenges to be solved before these innovative vaccination strategies may be used in tumor immunotherapy, developments in materials science and biotechnology might facilitate this process.

## Author contributions

**Conceptualization:** Hengheng Dai.

**Supervision:** Hengheng Dai.

**Writing – original draft:** Hengheng Dai.

**Writing – review & editing:** Hengheng Dai, Weiwei Kong, Tingting Liu, Xiaoxia Fei.
